# Supplementary material for: Did Homocysteine Take Part in the Start of the Synthesis of Peptides on the Early Earth?
Source: Biomolecules. 2022 Apr 8;12(4):555. doi: 10.3390/biom12040555 (PMC9031595; doi:10.3390/biom12040555)
Supplement: Supplementary file 1 [file biomolecules-12-00555-s001.zip › biomolecules-1669836-supplementary.pdf]

Did homocysteine took part in the start of the synthesis of peptides on the early Earth?

Sparta Youssef-Saliba, Anne Milet and Yannick Vallée*
yannick.vallee@univ-grenoble-alpes.fr

Supplementary Materials

Openings of thiolactones by amino acids, supplementary examples p. 2

Representative NMR spectra p. 4

Details for theoretical calculations p. 7

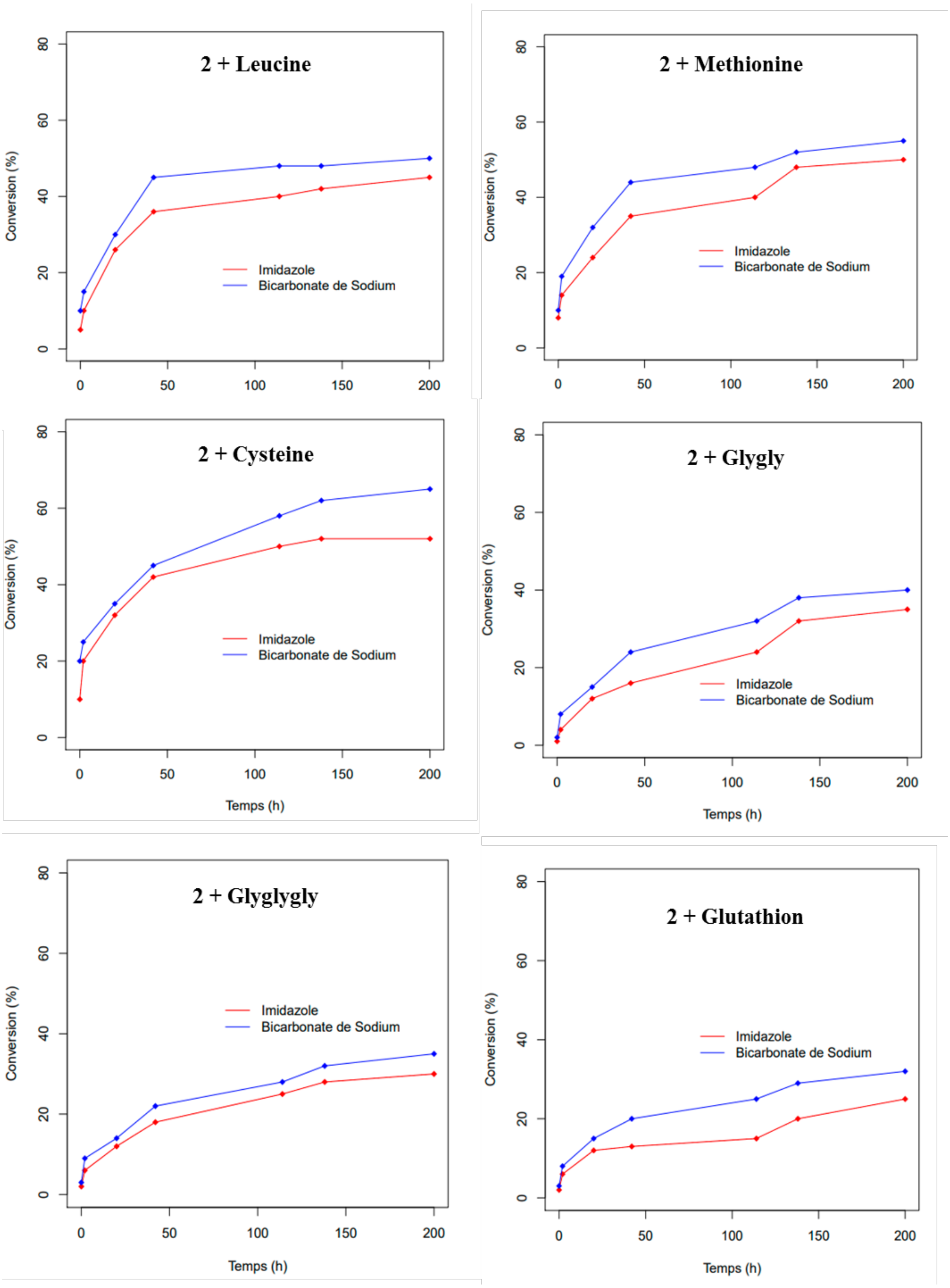

Figure S1: Evolution of peptide synthesis using homocysteine thiolactone and amino acids. Supplementary examples.

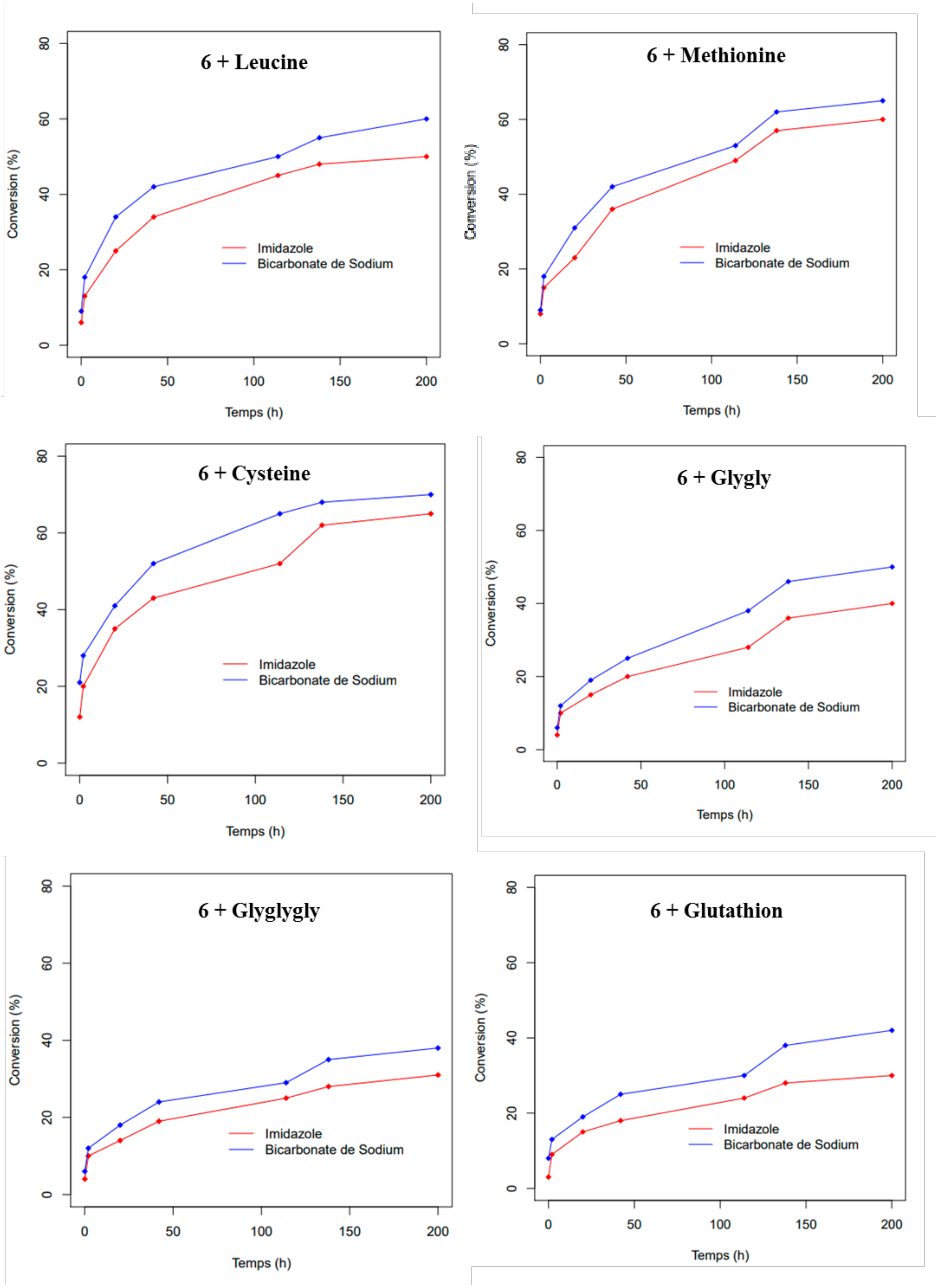

Figure S2: Evolution of peptide synthesis using *N*-Acetyl-homocysteine thiolactone and amino acids. Supplementary examples.

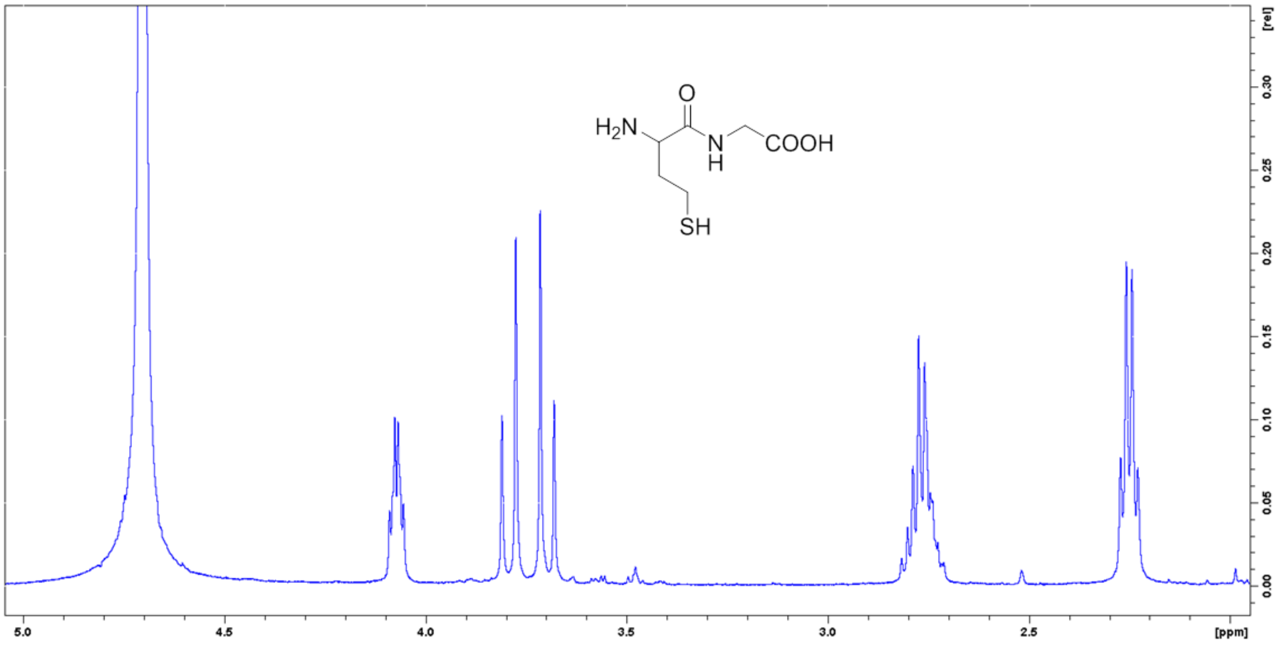

Figure S3: ^{1}H NMR spectra of Hcy-Gly, 500MHz, D_2O

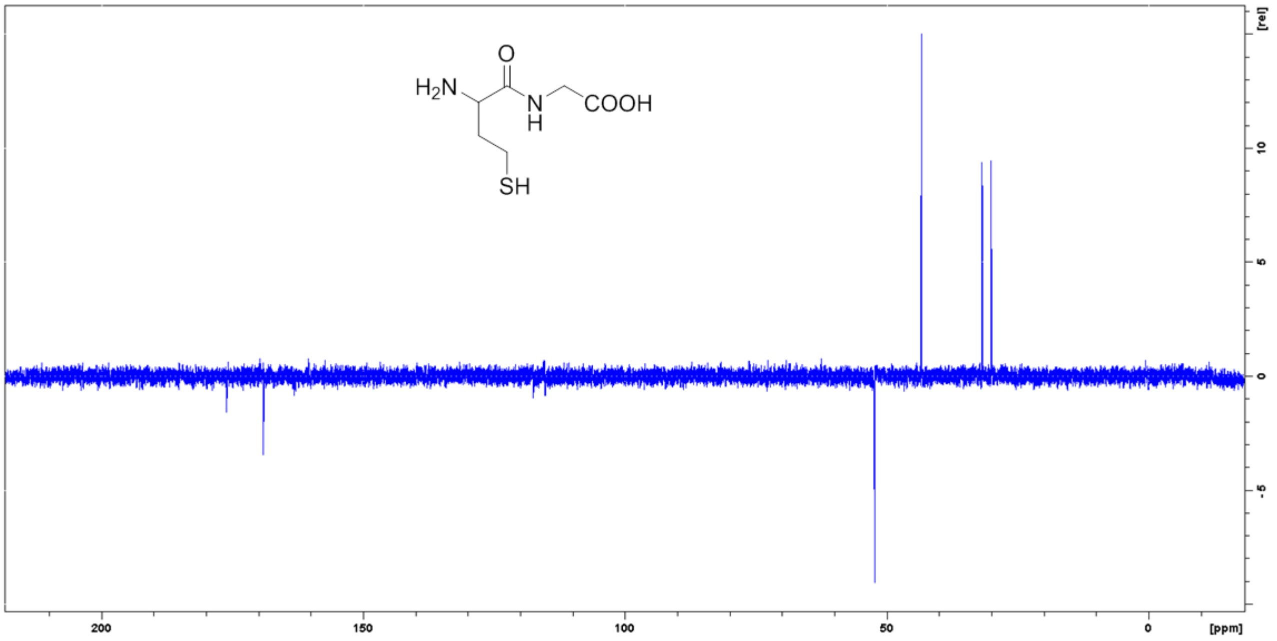

Figure S4: ^{13}C NMR spectra of Hcy-Gly, 125MHz, D_2O

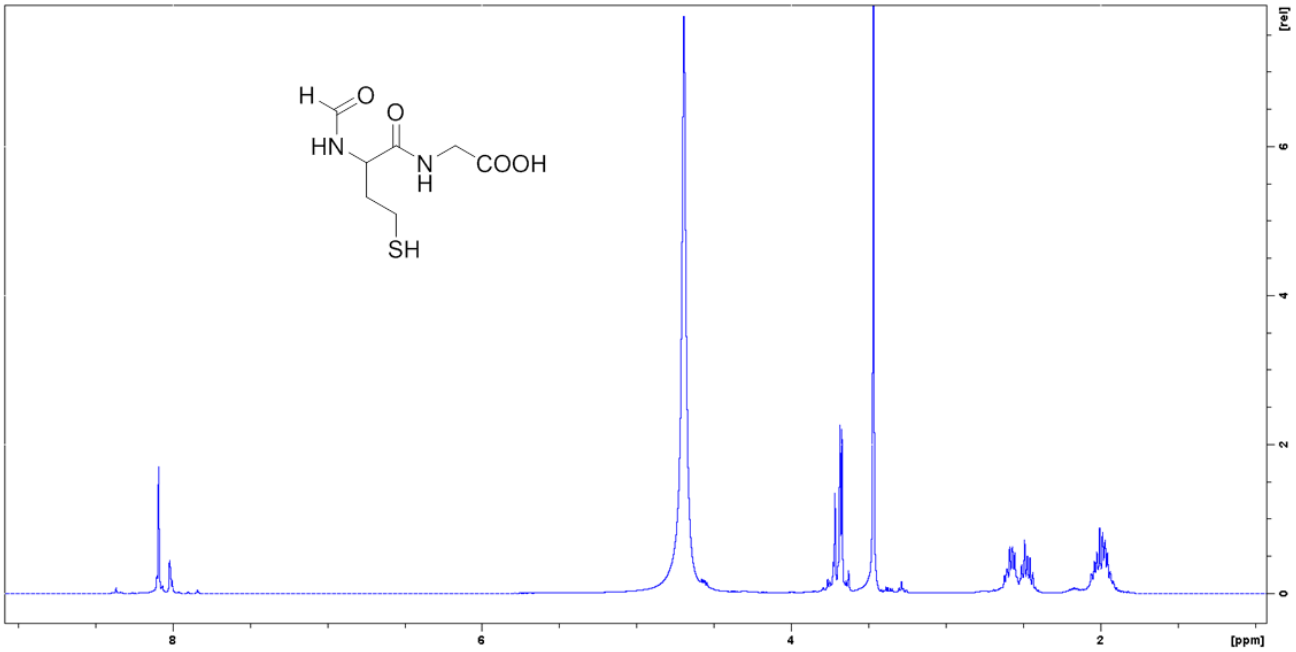

Figure S5: ^{1}H NMR spectra of *N*-formyl-Hcy-Gly, 500MHz, D_2O

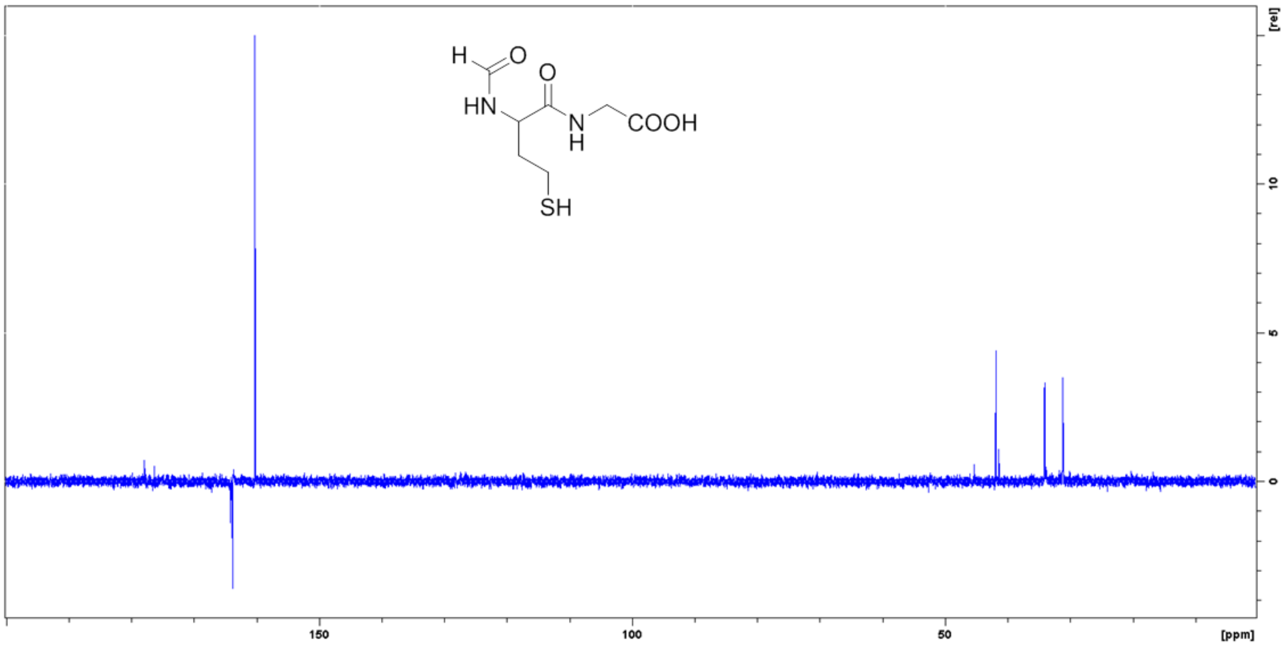

Figure S6: ^{13}C NMR spectra of molecule *N*-formyl-Hcy-Gly, 125MHz, D_2O

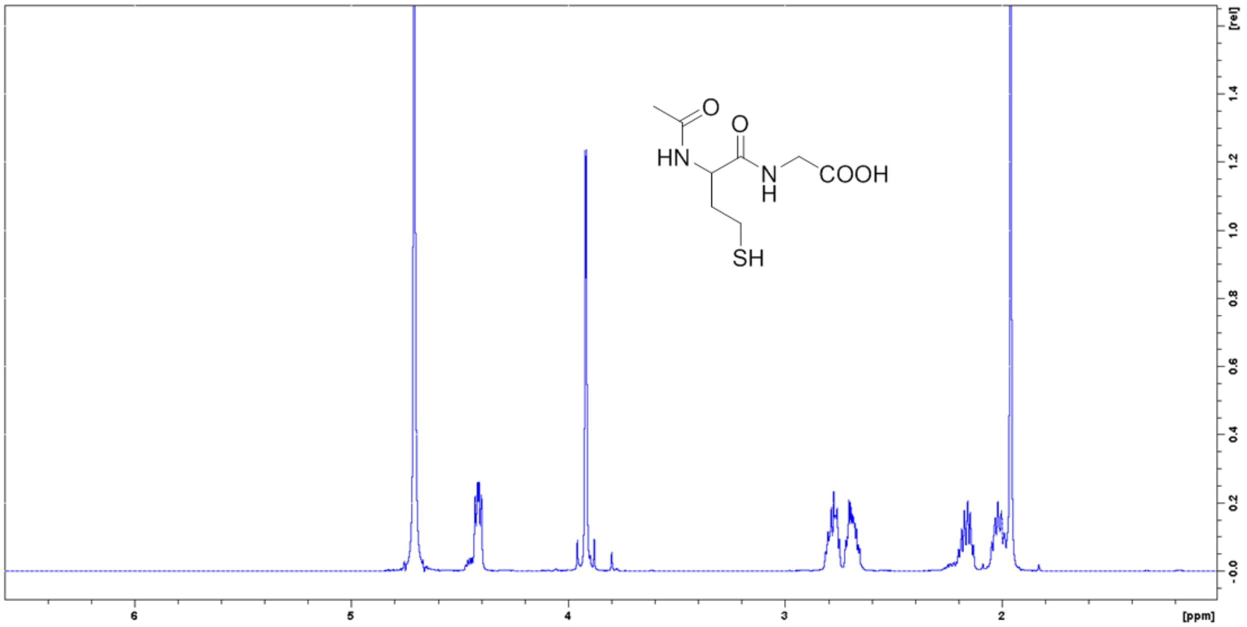

Figure S7: ^{1}H NMR spectra of *N*-acetyl-Hcy-Gly, 500MHz, D_2O

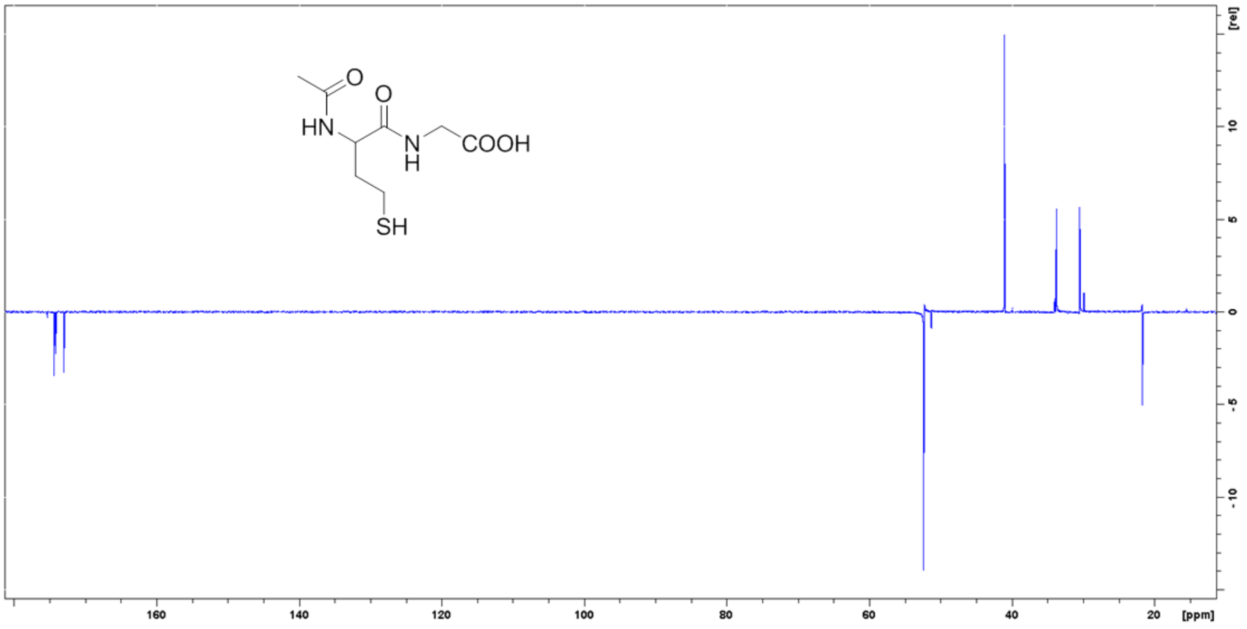

Figure S8: ^{13}C NMR spectra of *N*-acetyl-Hcy-Gly, 125MHz, D_2O

The reaction shown Scheme 3 was computed at different levels, the results are given in the Tables S1 and S2. Table S2 gives the results for the protonated version mentioned in the main article.

Level used	ΔE(kcal.mol^{-1})	ΔH(kcal.mol^{-1})	ΔG(kcal.mol^{-1})
B3LYP/def2TZVP	+3.20	+4.00	+0.11
B3LYP-D3BJ/def2TZVP	+3.28	+3.74	+3.31
B3LYP/6-31G**	+8.39	+9.42	+7.49
B3LYP-D3BJ/6-31G**	+9.17	+10.15	+7.53

Table S1: Energy difference corresponding to the reaction of Scheme 3. The energy are given in kcal.mol^{-1}.

Level used	ΔE(kcal.mol^{-1})	ΔH(kcal.mol^{-1})	ΔG(kcal.mol^{-1})
B3LYP/def2TZVP	-8.17	-7.50	-6.21
B3LYP-D3BJ/def2TZVP	-7.77	-7.35	-6.06

Table S2: Energy difference corresponding to the reaction of Scheme 3 involving H_3O^+-H_2O . The energy are given in kcal.mol^{-1}.

Level used	ΔE(kcal.mol^{-1})	ΔH(kcal.mol^{-1})	ΔG(kcal.mol^{-1})
B3LYP/def2TZVP	-9.26	-10.31	-16.12
B3LYP-D3BJ/def2TZVP	-6.31	-7.58	-10.56

Table S3: Energy difference between the diol and 2 corresponding to the reaction of Scheme 4. The energy are given in kcal.mol^{-1}.

Level used	$\Delta E^{\ddagger}$ (kcal.mol^{-1})	$\Delta H^{\ddagger}$ (kcal.mol^{-1})	$\Delta G^{\ddagger}$ (kcal.mol^{-1})
B3LYP/def2TZVP	-15.07	-11.44	-14.09
B3LYP-D3BJ/def2TZVP	-14.76	-10.97	-12.27

Table S4: Activation energy difference corresponding to the reaction of the diol to 2 shown Scheme 4. The energy are given in kcal.mol^{-1}.

Set up of the QM/MM part

Following optimisation at the B3LYP/6-31+G** level, single point energy calculation at the HF/6-31G* using the optimized geometry was performed to obtain partial charge on each atom with the restrained electrostatic potential (RESP) method [1]. The force fields parameters for the molecule were then deduced using the « gaff » parameters [2] with amber18 [3]. The complex was then immersed in a cubix box of TIP3P [4,5] waters adding 1546 molecules of solvent and two chloride anions to ensure the electric neutrality of the solution.

Equilibration involved an energy minimization, followed by a 25 ps of NVT dynamics at 300 K and finally a NPT dynamics to finally obtained a density around 1.02 and a cubic box size of 35.9084781 36.0573993 35.0994893 Å. The restraint on the homocysteine molecule during this process was of 100 kcal $Å^{-2}$ and 200 kcal $Å^{-2}$ for the NPT calculation.

Then starting from this structure, QM/MM dynamics were carrying out using the CP2K program. The QM part involves **1** and the surrounding water molecules at a distance of 6 Å (a molecule of **1** in 79 water molecules). Cf picture below. Using this tailored quantum part made of the molecule and a reasonable number of molecules around give the opportunity to the protons to move from **1** to the solvent while remaining close to the molecule. The objective is to keep the "acidic" aspect of the solution close to the molecule, this acidic aspect being a prerequisite for the reactivity. Another solution would have been to carry out purely quantum simulations, which would have required a smaller water box and the proton would have been able to sample the whole water box, which would have greatly lengthened the simulation without any gain from a chemical point of view since the objective is indeed to simulate the cyclization of **1**.

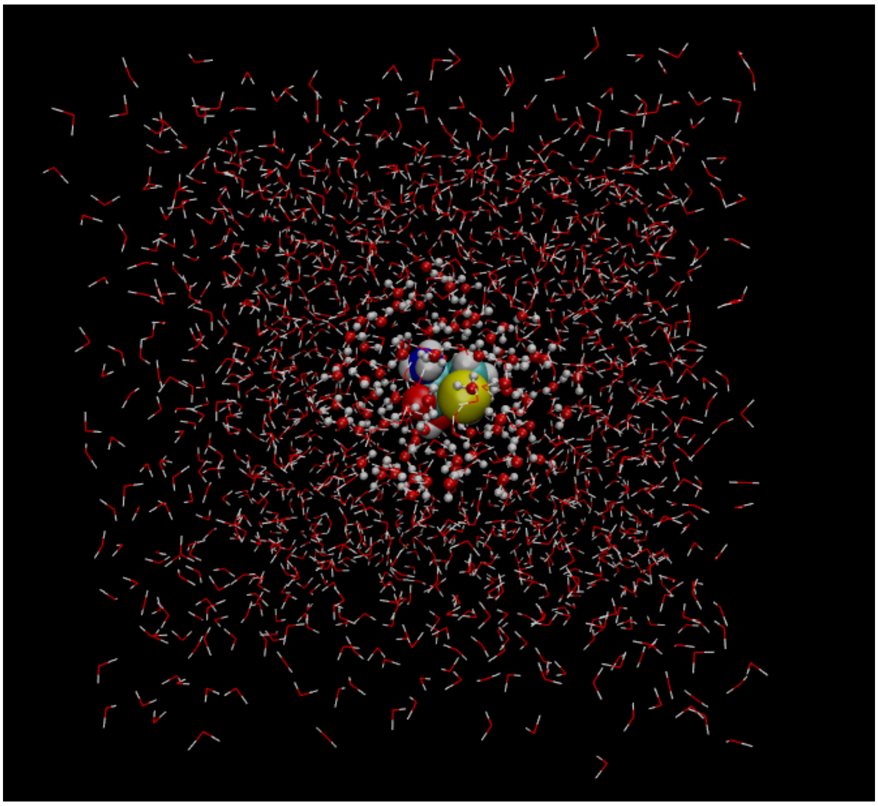

The ab initio Born-Oppenheimer dynamics calculations were performed using the CP2K- program at the DFT level with the BLYP functional with additional D3 dispersion corrections[6]. The basis set used was a double-ζ valence set of Gaussian orbitals with a set of polarization added for all atoms, the DZVP-MOLOPT-SR-GTH basis set [7] in conjunction with the Goedecker-Teter-Hutter pseudopotentials [8]. The auxiliary PW basis set was defined by a cubic box of 30 Å and by a density cutoff of 400 Ry.

The NVT dynamics at 300K using a CSVR thermostat (timecon of 70) and steps of 0.5 fs logically and rapidly induced a proton transfer from the carboxylic function to the surrounding water molecules. This type of structure (after the proton transfer) was used to perform the metadynamics simulations.
Metadynamics has been used to overcome the problem of observing rare events in conventional molecular dynamics and of finding the reaction coordinate. A series of small repulsive Gaussian potentials centered on the values of some collective variables (CV) are added during the dynamics, preventing the system from revisiting the same points in configurational space and creating a historydependent multidimensional biasing potential. A time step of 0.5 fs is used for the dynamics, and the hills of 1kcal/mol were added every 20 fs.
To study this reactivity three collective variables were used and all three are bonds distances. The first one is the carbon surfur bon to be formed, the second the S-H to be broken and finally the C-O to be broken. The width of the Gaussian used is 0.3 bohr and a reflective wall at 6.5 bohr was used to prevent sampling the C-S bond at large values which is of no interest since we study the cyclisation. The barrier is estimated to be 25kcal/mol.

Set up for the simulation of 1 in water, molecular dynamics
Following optimisation at the B3LYP/6-31+G** level, single point energy calculation at the HF/6-31G* using the optimized geometry was performed to obtain partial charge on each atom with the restrained electrostatic potential (RESP) method [1]. The force fields parameters for the molecule were then deduced using the « gaff » parameters [2] with amber18 [3]. The complex was then immersed in a cubix box of TIP3P [4,5] waters adding 2410 molecules of solvent and one chloride anions to ensure the electric neutrality of the solution. Equilibration involved an energy minimization, followed by a 25 ps of NVT dynamics at 300 K and finally a NPT dynamics to finally obtained a density around 1.02 and a cubic box size of 41.8510605 41.1527562 40.9236623 Å. The density obtained was around 1.011. Then these data were used to performed molecular dynamics in the NVT ensemble at 300K during 80 ns, the first 10 ns were taken as additional equilibration and the remaining 70 ns as production run.

Coordinates optimized at the B3LYP/def2TZVP/D3BJ with solvent effect (smd).

1 with five water molecules and one hydronium

```
   34
scf done: -1144.748841
 S    -0.733853    -2.644055     0.034966
 H    -0.875224    -3.146728    -1.205054
 C     1.085160    -2.424494    -0.019126
 H     1.353781    -2.119229     0.992706
 H     1.541713    -3.398335    -0.186858
 C     1.612797    -1.453795    -1.070806
 H     2.703844    -1.524004    -1.067475
 H     1.285501    -1.762557    -2.063767
 C     1.272983     0.035571    -0.914536
 H     1.805240     0.564620    -1.706962
 C    -0.186115     0.400980    -1.101911
 O    -0.836393     1.007522    -0.266706
 O    -0.635784     0.060966    -2.297911
 H    -1.567910     0.323985    -2.402982
 N     1.757798     0.599992     0.372293
 H     1.679894     1.637234     0.361462
 H     1.219881     0.255328     1.191097
 H     2.757357     0.347417     0.511063
 O    -3.324734     1.771520    -0.387577
 H    -3.930745     1.108545     0.183166
 O    -4.712975     0.250266     1.006466
 H    -5.426442     0.748541     1.431355
 H    -5.149728    -0.429927     0.473270
 H    -3.666335     1.819663    -1.295833
 H    -2.365843     1.447254    -0.414398
 O     0.387725    -0.149295     2.807024
```

```
 H     0.648539    -1.054074     3.025795
 O     1.512787     3.452690     0.234698
 H     1.715207     3.836477     1.098620
 O     4.491169    -0.155280     0.808322
 H     5.094422     0.553310     0.546478
 H     4.657446    -0.866499     0.174694
 H     0.563484     3.595117     0.119042
 H    -0.557797    -0.206540     2.613476
```

Diol (see Scheme 4 in main text) with explicit water molecules

```
   33
scf done: -1144.354347
 S    -0.781743    -1.391624     0.846323
 C     0.790192    -2.250085     0.444964
 H     1.518951    -2.037929     1.225230
 H     0.608293    -3.321397     0.415887
 C     1.233861    -1.708689    -0.910834
 H     2.294203    -1.891220    -1.084197
 H     0.680187    -2.202695    -1.710091
 C     0.912389    -0.225704    -0.960125
 H     0.974079     0.169009    -1.971117
 C    -0.537483    -0.043208    -0.455527
 O    -0.727100     1.218413     0.049359
 O    -1.455175    -0.222631    -1.518768
 H    -1.375845    -1.118743    -1.878205
 N     1.866830     0.572707    -0.135645
 H     1.731076     1.577707    -0.333227
 H     1.739795     0.434074     0.883856
 H     2.842914     0.316117    -0.387439
 H    -1.604739     1.291548     0.526088
 O     1.019352     3.252520    -0.914419
 H     1.192752     3.877809    -0.198567
 O     4.530153    -0.143711    -0.835304
 H     4.516256    -1.109176    -0.886759
 O     1.346076     0.325429     2.702047
 H     1.915419    -0.363191     3.071125
 H     0.181509     2.822124    -0.670679
 H     4.650828     0.148029    -1.749141
 H     0.496630    -0.121566     2.541433
 O    -3.052080     1.410399     1.292859
 H    -3.590514     0.822400     0.713054
 H    -3.012138     0.968183     2.150834
 H    -3.284369    -0.254603    -1.015555
 O    -4.179516    -0.192943    -0.629436
 H    -4.354515    -1.072416    -0.269765
```

Compound **2** with six water molecules

```
  33
scf done: -1144.364415
 S    -0.899865    -1.137880     1.148453
 C     0.446317    -2.252745     0.608660
 H     1.213933    -2.258990     1.379073
 H     0.039033    -3.255107     0.506307
 C     0.946302    -1.694953    -0.724457
 H     1.962495    -2.030115    -0.928380
 H     0.305863    -2.056832    -1.528029
 C     0.850517    -0.180952    -0.698709
 H     0.698280     0.239189    -1.689916
```

```
 C    -0.361155     0.202350     0.163085
 O    -0.865609     1.310137     0.162598
 O    -2.236198    -0.347241    -2.640930
 H    -1.532591    -0.958780    -2.394223
 N     2.064127     0.477008    -0.131845
 H     1.947374     1.506732    -0.182832
 H     2.234068     0.216209     0.860844
 H     2.898671     0.209977    -0.696670
 H    -2.545790     1.555622     0.913234
 O     1.285209     3.234710    -0.436693
 H     1.479403     3.727405     0.371661
 O     4.295510    -0.273442    -1.683446
 H     4.267447     0.241681    -2.501134
 O     2.546214    -0.033156     2.621515
 H     3.501644     0.039541     2.749962
 H     0.383527     2.896092    -0.310322
 H     5.084958     0.040385    -1.221797
 H     2.329207    -0.935556     2.890726
 O    -3.495747     1.534750     1.138598
 H    -3.964860     0.212211     0.024235
 H    -3.534210     1.211236     2.047850
 H    -2.881034    -0.396117    -1.901530
 O    -4.177765    -0.477013    -0.644815
 H    -4.028039    -1.317914    -0.194739
```

TS from diol to **2**

```
   33
scf done: -1144.330827
 S    -0.712329    -1.620232     0.197410
 C     0.809320    -2.183551    -0.655452
 H     1.567849    -2.407952     0.092810
 H     0.593636    -3.090277    -1.215112
 C     1.245218    -1.044251    -1.577587
 H     2.301732    -1.130967    -1.832306
 H     0.683174    -1.084536    -2.511008
 C     0.949607     0.281325    -0.903336
 H     0.970196     1.107800    -1.608636
 C    -0.456180     0.222757    -0.249452
 O    -0.741622     1.059664     0.671552
 O    -1.427342     0.495576    -1.471171
 H    -1.339494    -0.194144    -2.149330
 N     1.953065     0.605516     0.155167
 H     1.808601     1.576962     0.472512
 H     1.870570    -0.006783     0.987720
 H     2.912092     0.513964    -0.234910
 H    -2.187272     0.905846     1.514465
 O     0.871014     3.236234     0.833970
 H     1.029441     3.396345     1.772877
 O     4.548077     0.258956    -0.972795
 H     4.605752    -0.705553    -1.012093
 O     1.469792    -0.941991     2.548098
 H     2.041665    -1.720538     2.581718
 H     0.148039     2.566454     0.807354
 H     4.434774     0.530997    -1.893805
 H     0.632343    -1.268899     2.170273
 O    -3.132743     0.844425     1.820654
 H    -3.646141     0.607175     0.304887
```

```
H    -3.191363     0.023897     2.327252
H    -2.557693     0.490078    -1.109430
O    -3.725503     0.531864    -0.700338
H    -4.162983    -0.310972    -0.891668
```


1. Bayly, C.I.; Cieplak, P.; Cornell, W.; Kollman, P.A. A Well-Behaved Electrostatic Potential Based Method Using Charge Restraints for Deriving Atomic Charges: The RESP Model. *J. Phys. Chem.* **1993**, *97*, 10269–10280, doi:10.1021/j100142a004.
2. Wang, J.; Wolf, R.M.; Caldwell, J.W.; Kollman, P.A.; Case, D.A. Development and Testing of a General Amber Force Field. *Journal of Computational Chemistry* **2004**, *25*, 1157–1174, doi:https://doi.org/10.1002/jcc.20035.
3. *Amber18*; Case, D.A; Ben-Shalom, I.Y.; S.R. Brozell; Cerutti, D.S. *et al*. AMBER 2018, University of California, San Francisco.
4. Hutter, J.; Iannuzzi, M.; Schiffmann, F.; VandeVondele, J. Cp2k: Atomistic Simulations of Condensed Matter Systems. *WIREs Computational Molecular Science* **2014**, *4*, 15–25, doi:https://doi.org/10.1002/wcms.1159.
5. Kühne, T.D.; Iannuzzi, M.; Del Ben, M.; Rybkin, V.V.; Seewald, P.; Stein, F.; Laino, T.; Khaliullin, R.Z.; Schütt, O.; Schiffmann, F.; et al. CP2K: An Electronic Structure and Molecular Dynamics Software Package - Quickstep: Efficient and Accurate Electronic Structure Calculations. *The Journal of Chemical Physics* **2020**, *152*, 194103, doi:10.1063/5.0007045.
6. Grimme, S.; Antony, J.; Ehrlich, S.; Krieg, H. A Consistent and Accurate Ab Initio Parametrization of Density Functional Dispersion Correction (DFT-D) for the 94 Elements H-Pu. *J. Chem. Phys.* **2010**, *132*, 154104, doi:10.1063/1.3382344.
7. VandeVondele, J.; Hutter, J. Gaussian Basis Sets for Accurate Calculations on Molecular Systems in Gas and Condensed Phases. *J. Chem. Phys.* **2007**, *127*, 114105, doi:10.1063/1.2770708.
8. Goedecker, S.; Teter, M.; Hutter, J. Separable Dual-Space Gaussian Pseudopotentials. *Phys. Rev. B* **1996**, *54*, 1703–1710, doi:10.1103/PhysRevB.54.1703.
